# Implementation science evaluation of an eHealth pediatric primary-care overweight and obesity intervention using the RE-AIM evaluation framework

**DOI:** 10.1371/journal.pone.0341635

**Published:** 2026-02-09

**Authors:** Joshua S. Yudkin, Rebecca M. Jungbauer, Marlyn A. Allicock, Sarah E. Barlow

**Affiliations:** 1 School of Public Health, Texas A&M University, College Station, Texas, United States of America; 2 Pacific Northwest Evidence-based Practice Center, Oregon Health and Science University, Portland, Oregon, United States of America; 3 School of Public Health, University of Texas Health Sciences Center at Houston, Houston, Texas, United States of America; 4 Children’s Health, Dallas, Texas, United States of America; 5 Department of Pediatrics, University of Texas Southwestern Medical Center, Dallas Texas United States of America; The Chinese University of Hong Kong, HONG KONG

## Abstract

**Introduction:**

The childhood overweight and obesity (OW/OB) epidemic in the United States disproportionately affects children from marginalized backgrounds, particularly those who lack the financial resources or support to engage in evidence-based interventions. Moreover, healthcare systems struggle to deliver these interventions equitably. To address this gap, this study evaluated the implementation science outcomes of Dynamo Kids! (DK!), a novel primary-care-based eHealth intervention intended for families with children ages 6–12 with OW/OB.

**Materials and methods:**

DK! was developed within a major safety-net hospital system in Dallas, TX. Ten providers in three different clinics were instructed to offer DK! to 581 families with children ages 6–12 with OW/OB. Guided by the Reach, Effectiveness, Adoption, Implementation Maintenance (RE-AIM) framework, we evaluated the implementation science outcomes of the DK! pilot conducted between August 2020 and April 2021, using eight different data sources—both qualitative and quantitative.

**Results:**

Building on previously published findings that demonstrated an increase in Family Nutrition Physical Activity score and a decrease in child%BMIp95, this study engaged 46 families and ten providers with diverse backgrounds in a safety-net practice who, generally, self-reported improved knowledge, skills, and self-efficacy in treating overweight and obesity. Most parents were satisfied with the DK! intervention, using the website with high variability, on average for two hours 24 minutes of total user time over three months and endorsed the patient-centered care in qualitative interviews. Both families and providers generally found DK! to be important, trustworthy, tailored, and accessible and endorsed the DK! intervention.

**Discussion:**

DK! demonstrated limited success in its initial setting, with strong support for its continuation and expansion from both the healthcare system and patient families. Future studies should incorporate user recommendations and consider comparing outcomes to a control group beyond the context of the COVID-19 pandemic.

## Introduction

### Childhood Obesity

The childhood overweight and obesity (hereafter “OW/OB”) pandemic [1] is a significant health challenge that is also associated with numerous other diseases including hypertension, diabetes mellitus, sleep disorders, liver diseases, and cancer [2–6]. In pediatric populations worldwide, the prevalence of OW/OB has increased fourfold since 1990, with an estimated 20% of children aged five to 19 years living with OW/OB in 2022 [7]. Similarly, in the United States (US), the prevalence of OW/OB among children aged 2–19 years has nearly quadrupled over the past 50 years, rising from 5% to 19.7% in 2020 [8–12]. Recent estimates from 2024 indicate that 21% of adolescents in the United States are living with obesity [6].

Obesity disproportionately affects populations that are underserved and/or under-resourced, including racial and ethnic minority groups and low-income households [8,10]. During and since the COVID-19 pandemic, children have experienced accelerated weight gain compared to pre-pandemic levels [13,14], further exacerbating existing health disparities and outcomes [15]. This situation has created renewed urgency to address childhood obesity as a key component of the post-pandemic recovery efforts [16].

Recommendations from the US Preventive Services Task Force and other organizations highlight the effectiveness of comprehensive, family-based, moderate- to high-intensity interventions, involving at least 26 contact hours over two to 12 months in promoting weight loss [13,15–17]. However, several challenges within the US healthcare system hinder the delivery of these evidence-based OW/OB interventions, particularly to populations at highest risk.

For families, clinical visits can be challenging due to conflicts with school and work schedules, communication barriers related to cultural differences or limited health literacy, and insufficient insurance coverage for necessary care.^12^ Similarly, providers may lack the knowledge, skills, and time to effectively address these issues within the demanding environment of busy primary care settings.^11^

Aligned with the World Health Organization’s global strategy on digital which posits that eHealth interventions [18] strengthen health systems and support universal health coverage rather than leaving patients behind [19], an eHealth intervention may offer an equitable strategy to overcome the provider- and patient-level barriers to obesity care mentioned above. In fact, eHealth interventions have been documented to advance equity and improve health care access and disease management, often at a reduced cost [20–22]. However, existing research in weight management has focused primarily on mobile alerts, digital newsletters, and telehealth engagement in adults, rather than on personalized, self-guided online programs for children. In adult populations [23], research demonstrates that online health promotion efforts for managing OW/OB have proven both feasible and effective in promoting positive behavior change and addressing access barriers [24–26]. Adult participants have reported that eHealth technologies [18] provide engaging, individualized support for behavior change, with interventions being viewed as feasible, acceptable, accessible, and effective across various eHealth strategies and populations [24,25,27]. However, few studies have piloted [17,28,29] or fully implemented technology-based solutions for pediatric OW/OB treatment, especially in primary care settings and pediatric populations [26,30,31]. There remains a critical need to evaluate the effectiveness of eHealth interventions in pediatric care and primary care contexts.

According to the Obesity Chronic Care Model (OCCM) framework [23,32], parents and healthcare providers have complementary yet distinct roles in the treatment of childhood OW/OB. The proactive healthcare provider initiates a relationship with the family, focuses on the child’s health condition, delivers a clinical diagnosis, and communicates that OW/OB are recognized medical conditions. In turn, the engaged parent supports the child’s health by implementing healthy environmental and behavioral changes at home.^11^

Informed by the complementary approach of the OCCM [23,33,34], a quasi-experimental, family-based, self-paced, and personalized pilot intervention called Dynamo Kids! (DK!) was developed for parents and caregivers of pediatric patients ages 6–12 years with OW/OB to be offered by pediatric primary care providers with the aim of improving family-level obesity prevention behaviors as well as BMI outcomes in both children and parents. The rationale, development, and preliminary effectiveness of DK! have been described in previous publications [4,17,28,35]. DK! is a low-cost, scalable program that may help address the significant delays in the dissemination and implementation of effective interventions.

### Use of the RE-AIM framework to evaluate an online pediatric obesity program

The RE-AIM (Reach, Effectiveness/Efficacy, Adoption, Implementation, and Maintenance) framework is one of the most frequently used tools in implementation science for planning, describing, and evaluating community- and public health-based behavior change interventions [36–41]. The RE-AIM dimensions were originally developed to address delays in the translation of research evidence into practice [42] and are measured across multiple levels, settings (including digital and eHealth platforms), and populations [43,44]. Over time, the framework has evolved, with recent applications helping researchers identify key dimensions to improve intervention uptake and sustainability [42]. The aim of this pilot study was to evaluate the DK! program using the RE-AIM framework to identify elements that would facilitate its implementation and adoption in pediatric settings.

## Materials and methods

The content, design, and initial findings from the DK! needs assessments and effectiveness study have been described previously [4,17,28,35]. Briefly, informed by the OCCM [23,33,34] and aligned with stakeholder preferences [45], DK! was implemented in a safety-net healthcare system and featured two clinic-based components for healthcare systems and providers: 1) an electronic health record (EHR)-embedded best practice alert (BPA) to notify providers when a patient meets DK! inclusion criteria, and 2) a customized report for providers, including talking points for use during follow-up visits with patient families. The BPA required providers to select one of four options before continuing to access the patients’ file: families were not suitable candidates, families were offered the intervention and accepted, families were offered the intervention and declined to participate, or the provider dismissed the alert. Families were recruited from August 1^st^, 2020 to April 30^th^, 2021.

Additionally, DK! included a virtual portal for patient caregivers (referred to as “parents”) to access a customized, self-paced bilingual (English and Spanish) educational website focused on reducing sugar-sweetened beverage consumption, increasing physical activity, and promoting adherence to MyPlate nutritional guidelines. Each module consisted of four parallel sections: “Learning More”, which focused on knowledge acquisition, “Building Skills”, which provided strategies for facilitating behavioral change, “Barriers”, which addressed common challenges related to these behavior change(s), and “Moving Forward”, which encouraged reflection and reinforcement of the previous three weeks. While the modules followed a standard structure, parents could choose the order in which they engaged with each topic.

Key programmatic features included module-specific goal-setting and tracking, a resource library with both in-person and virtual information and activities, and low-cost healthy recipes from the U.S. Department of Agriculture’s MyPlate program. Additionally, housed in the “Ask the Doctor” subsection of each module, the website included real patient stories from a variety of backgrounds and family configurations to help families make changes. The website was written with accordion formatting to allow families to gain more or less information based on their preferences. Finally, it was structured to allow parents to engage with the website for several hours a week, planned that way based on pre-production feedback. In this study, parents were able to complete asynchronous weekly activities on the DK! website for three months [35,46].

Families were evaluated both before exposure to any content and after completing the intervention. The modules included built-in time delays that encouraged families to focus on a single topic for one full week before advancing to the next module. Prior to the intervention’s launch, providers received a one-hour orientation to DK! along with training in motivational interviewing [47], the training focused on best practices for engaging families in weight management discussions.

The BPA identified eligible families, and the provider’s response to the BPA generated a list of patients who were willing to be contacted, which was sent to the research team: 100 parents provided verbal consent through a research assistant [17]. Seventy-three parents completed the baseline survey (referred to as “engaged”) [28], and 46 families accessed at least one topic on the DK! website (referred to as “users”).

Importantly, mixed-methods follow-up data were collected from both users (n = 46) and non-users (n = 27) through surveys and post-intervention interviews to assess potential selection bias. Not all participants completed the follow-up surveys in full: 33 users (72%) and nine non-users (33%) completed the Family Nutrition and Physical Activity (FNPA) survey section, 30 users (65%) and six non-users (22%) self-reported BMI changes, and 15 users (33%) responded to questions about their DK! experience.

This study was reviewed and approved by the Institutional Review Boards of both UTHealth (HSC-SPH-19–0660) and UT Southwestern/Parkland (STU 2019−0876) and was developed in accordance with the Strengthening the Reporting of Observational Studies in Epidemiology (STROBE) guidelines.^29^

### Sample population and study setting

This pre-post cohort pilot eHealth study was conducted at three primary-care clinics within a safety-net hospital system in Dallas, TX. The target population consisted of pediatric patients ages 6–12 years with a BMI ≥ 85th percentile, along with their parents, all of whom resided in low socioeconomic ZIP codes and spoke English and/or Spanish.

### Data sources

This evaluation was conducted using eight different data sources.

1. *Electronic Health Record (EHR):* Socio-demographic data, clinical values, well-child visit information, and past medical history were all stored in Epic software.
2. *Research Team and Study Protocol:* The research team provided feedback on the adaptation of *a priori* processes during the pilot study and in interactions with study participants.
3. *Pre-Post Participant Survey:* A 105-item baseline survey and a 114-item follow-up survey, along with additional process questions, were administered via Qualtrics to DK! participants before their engagement with the DK! website and after the three-month study period (Appendices 1 and 2).
4. *Backend Data from the DK! Website*: Within the DK! website, patients had the opportunity to set personalized goals using a goal-tracking tool and report their progress. Additionally, at the end of each of the three modules, 11 questions assessed both the parent experience and self-reported changes in family behaviors.
5. *Pre-Post Provider Survey:* Ten providers at three clinics completed a 57-item survey before and after the DK! pilot to assess provider knowledge, attitudes, and behaviors regarding the treatment of overweight and obesity (S3 Appendix).
6. *Intervention Implementation:* A research team at the health system designed, implemented, and delivered DK! in the clinical setting, collaborating with both clinical and non-clinical partners.
7. *Provider Fidelity Checklist:* As part of the process evaluation, providers were asked to complete this survey tool at the end of each follow-up visit with DK! patients (S4 Appendix).
8. *Post-intervention Participant Qualitative Interviews and Provider Focus Groups*: All patients who completed the baseline survey—regardless of whether they used the DK! website—were invited to participate in a follow-up qualitative interview with a researcher. 19 parents completed telephone interviews, including four non-users. Additionally, a semi-structured provider focus group was conducted at each clinic where DK! was offered, with nine of the ten providers participating.

### Measure Development

Aligned with previous weight management research using the RE-AIM framework [31,48,49], evaluation indicators were established for each of the RE-AIM constructs, focusing on implementation outcomes that assessed feasibility and identified characteristics that facilitate intervention uptake [42]. Table 1 presents the definitions and corresponding measurements for each RE-AIM construct, along with the associated data sources for each measurement/indicator.

**Table 1:** Defining the RE-AIM dimensions in the dynamo kids! (DK!) weight management program evaluation [36,48].

Definitions of the RE-AIM dimensions with exemplary measures used in the evaluation of the DK! Weight Management Program (definitions adapted from www.re-aim.org)
Exemplar measures and data source
**Reach** *Patient-Level:* 1. Total number of participants identified as eligible by the electronic health record (EHR) and providers, along with their socio-demographic characteristics^1^ 2. Total number of participants who provided consent, along with their socio-demographic characteristics^2^ 3. Total number of participants who completed the baseline survey, along with their socio-demographic characteristics^3^ 4. Total number of participants who participated in DK! program^4^
**Effectiveness** *Patient Level:* 1. Change in the FNPA survey score (primary outcome for effectiveness)^3^ 2. Change in Child %BMI_p95_ calculated from weight, height, sex, and age in months as measured by health professionals^1^ 3. Change in parent BMI based self-reported weight and height^3^ *Setting/Provider Level:* 1. Change in provider knowledge, attitudes, and behaviors regarding the treatment of overweight and obesity.^5^
**Adoption** *Patient Level:* 1. Percent of participants who used the DK! website (primary outcome for adoption)^4^ 2. Amount of time participants spent using the DK! website^4^ 3. Satisfaction, as measured by self-reported perceived importance, adoption of behaviors, and information sharing^3,4^ 4. Percent of patients who returned for a follow-up weight management visit^1^ *Setting/Provider Level:* 1. Willingness of the system to develop the tool and integrate it with the EHR for DK!^6^ 2. Total number of providers who offered DK! along with their socio-demographic characteristics^1^ 3. The proportion (and characteristics) of eligible patients to whom providers offered DK!^1^
**Implementation** *Patient Level:* 1. User report of experience, barriers, facilitators, and preferences^4^ 2. User recommendations^4^ *Setting/Provider Level:* 3. Fidelity to the *a priori* study protocol^2,7^ 4. Documenting challenges and unplanned implementation changes ^2,7^ 5. User experience, barriers, facilitators, and preferences^8^ 6. User recommendations^8^
**Maintenance** *Patent Level:* 1. Parent interest in program continuation^8^ *Setting/Provider Level:* 1. Program status at the time of manuscript submission^2^ 2. Stakeholders (providers, practice administration) interest in program continuation^8^ 3. Financial and staffing capacity to sustain DK!^2,8^

*Notes*:

1.All constructs were measured and supported by qualitative data collected from patient interviews and provider focus groups.

2.Data sources (superscripts) are described in more detail in the Methods section:

1. Abstract from the electronic health record (EHR)

2. Research team and study protocol

3. Pre-post participant survey

4. Back-end data from the DK! website

5. Pre-post provider survey

6. Intervention actualization

7. Provider fidelity checklist

8. Post-intervention participant interviews and provider focus groups

Briefly, “Reach” was assessed at the patient level, considering not only the total number of participants who continued through each phase but also their sociodemographic and clinical characteristics. “Effectiveness” was measured by the changes in FNPA survey scores (the study’s primary outcome), Child %BMIp95, parent BMI values, and provider behaviors during clinical visits. “Adoption” was evaluated at both the participant and setting levels. At the participant level, adoption was measured by the number of participants who used the DK! website, the extent and type of engagement, and overall satisfaction. At the setting level, adoption was assessed based on the system’s implementation of DK!, the total number of providers who offered DK!, and the proportion of eligible patients who were offered the program. “Implementation” referred to adherence to the a priori study protocol that was grounded in evidence-based practices, as well as the reported experiences of both providers and participants, measured at both the health system and patient levels. Finally, “Maintenance” was assessed at both levels, with a primary focus on the feasibility and interest in sustaining the DK! pilot intervention beyond the study period.

This study was approved by the Institutional Review Board at the University of Texas Southwestern Medical Center (UTSW) and the Committee for the Protection of Human Subjects at the UTHealth School of Public Health. All study procedures complied with institutional guidelines and the ethical principles outlined in the Declaration of Helsinki. Written informed consent was obtained from all participants prior to participation.

### Analysis

The methodology for the descriptive statistics applied to all quantitative variables has been described in previous publications [17,28]. For qualitative analysis, the Population Health Lab at UT Southwestern Medical Center transcribed and translated all interviews and focus groups with parents and providers. Two independent researchers conducted thematic analysis [50] on 22 patient interviews and three semi-structured provider focus groups, and then compared and reconciled their findings. Quantitative and qualitative data were triangulated to provide a more comprehensive understanding of the implementation science outcomes of the DK! intervention.

## Results

### Reach

At three primary care clinics, 581 patients met the eligibility criteria, as described in previous research [17,28]. Of these, 366 (63%) were excluded for the following reasons: (a) the provider determined the families were not suitable candidates (n = 170), (b) families declined to participate (n = 82), (c) the provider dismissed or ignored the alert for other patients (n = 73), or (d) no provider action was recorded in the EHR (n = 32). Of the 215 patients, 37% referred to DK!, 100 families (46.5%) consented, enrolled, and were included in the analysis.

The demographics of the study participants, including those who were excluded or lost to follow up, have been reported in previous publications [28]. There were no significant demographic differences among participating providers, most were female, and approximately half were Spanish-speaking (Table 2).

**Table 2:** Provider and clinic characteristics.

Provider Characteristics	n (%)
Provider Race	
Non-Hispanic White	3 (30)
Non-Hispanic Black	3 (30)
Asian American	3 (30)
Pacific Islander/Hawaiian	1 (10)
Number of Providers Who Can Communicate Directly with Patients in Spanish	
Spanish-Speaking	5 (50)
Provider Sex	
Female	9 (90)
Missing	1 (10)
Provider Training	
MD or DO	8
NP or PA	2
Providers’ Years in Practice	
Mean Number of Years (SD) (Range:3–27)	18.7 (6.7)
Number of Providers Offering DK! in the Clinic	
Clinic 1	3 (30)
Clinic 2	3 (30)
Clinic 3	4 (40)

*Note:* Clinic leadership has requested that provider data be analyzed at the aggregate level only, due to the relatively small number of providers in this pilot study.

The provider focus groups and patient interviews offered deeper insights into parent participation. Some parents consented to participate because they were excited about potential solutions for the “whole family” while others focused solely on finding solutions for the children with weight issues. Some parents recognized that their children were not only overweight but also had comorbidities such as diabetes or hypertension, while others were “surprised to learn their child was overweight.” In some families, parents stated that it was their “child who actually chose it [DK!]” after the provider suggested the program. In other families, the goal was for both parents and children to “do it [DK!] together and support each other through the program.”

### Effectiveness

The effectiveness of DK! on the primary outcome—pre-post changes in FNPA screening composite scores, as well as changes in parent and child BMI—has been reported previously. In summary, of the 100 families who consented, 73 completed the baseline survey, and 46 engaged with the DK! website, reporting an increase in FNPA scores and a decrease in child %BMIp95. In adjusted regression models, each additional minute spent on the DK! website was associated with a −0.02% change in child %BMIp95 [95% confidence interval: −0.03 to −0.01]. This pilot study was conducted during the COVID-19 pandemic, a period when rates of BMI increase nearly doubled compared to pre-pandemic trends [46].

Compared with baseline self-reported data, more providers (a) successfully identified the correct Centers for Disease Control and Prevention (CDC) definition of obesity, (b) became more aware of available community resources to share with patients, and (c) noted a 60% increase in clinical encounters for OW/OB by the end of the three-month DK! pilot intervention. Additionally, compared with self-reported baseline data, more providers reported (a) asking families for permission to discuss OW/OB (3.2 vs 3.5 on a 5-point Likert scale), (b) inquiring about which issues were important to address during visits (3.7 vs 4.4), (c) assessing family readiness for change (3.8 vs 4.3), (d) referring families to another specialty or program (3.1 vs 3.9), and (e) recommending online tools to treat OW/OB more frequently (2.1 vs 2.9). Providers also reported being less likely to believe that weight-related visits were not worthwhile (2.3 vs 1.6) (Table 3).

**Table 3:** Assessing effectiveness for healthcare providers using data from the provider survey.

Questions	Pre n (%)	Post n (%)
Correctly Identifying the CDC Definition of Pediatric Overweight	Baseline (n = 10)	Follow-up (n = 10)
Yes	10 (0)	9 (90)
No	0 (0)	0 (0))
Missing	0 (0)	1 (10)
Correctly Identifying the CDC Definition of Pediatric Obesity	Baseline (n = 10)	Follow-up (n = 10)
Correct (95th percentile and above)	7 (70)	8 (80)
Nearly correct (94th percentile and above)	3 (30)	1 (10)
Missing	0 (0)	1 (10)
We have community resources to support childhood healthy lifestyle (example recreation programs, farmers markets) in English	Baseline (n = 10)	Follow-up (n = 10)
Yes	8 (80)	5 (50)
No	1 (10)	4 (40)
Don’t know	1 (10)	0 (0)
Missing	0 (0)	1 (10)
We have community resources to support childhood healthy lifestyle (example recreation programs, farmers markets) in Spanish	Baseline (n = 10)	Follow-up (n = 10)
Yes	8 (80)	6 (60)
No	1 (10)	3 (30)
Don’t know	1 (10)	0 (0)
Missing	0 (0)	1 (10)
Number of encounters in the past 3 months for Overweight/Obesity (OW/OB) (provider recall)	Baseline (n = 10)	Follow-up (n = 10)
Mean number of encounters (SD)	10.8 (10.7)	17.4 (23.4)
Range of the number of encounters	2–30	3–70
How much do you agree with the following statements when seeing a child with obesity/overweight... Likert Scale Questions (1 Lowest, 5 Highest)	Baseline (n = 10)	Follow-up (n = 9)
Talking to a parent about a child’s overweight or obesity is an uncomfortable discussion	3.5 (1.2)	3.6 (0.9)
Parents often take offense when I discuss child’s overweight or obesity	3.2 (1.0)	3.3 (0.9)
Concern about parent’s reaction is a barrier to discussing child’s overweight or obesity	2.9 (1.4)	3.7 (1.1)
Futility or lack of effective intervention is a barrier to discussing overweight or obesity	2.3 (1.0)	2.8 (1.3)
With children with overweight and obesity, how often do you … Likert Scale Questions (1 Never, 5 Always)	Baseline (n = 10)	Follow-up (n = 9)
Evaluate medical conditions	4.7 (0.5)	5 (0.0)
Address nutrition	4.8 (0.4)	4.9 (0.3)
Address physical activity	4.8 (0.4)	4.9 (0.3)
Address screen time	4.6 (0.5)	4.7 (0.5)
Ask permission [to discuss their child’s weight]	3.2 (1.7)	3.9 (1.2)
Ask which issues are important	3.7 (1.3)	4.4 (0.7)
Assess readiness for change	3.8 (1.1)	4.3 (0.7)
Assess confidence [in ability to make changes]	3.6 (1.3)	3.9 (1.3)
Schedule a return visit for weight	4.2 (0.6)	3.9 (0.8)
Refer to dietitian	3.8 (1.0)	4.2 (0.4)
Refer to another specialty/program [to address overweight/obesity]	3.1 (1.2)	3.9 (0.6)
Recommend online tool	2.1 (1.2)	2.9 (1.1)
How much is ___ a barrier to scheduling visits specifically to address OW and OB?… Likert Scale Questions (1 Never, 4 Always)	Baseline (n = 10)	Follow-up (n = 9)
Lack of reimbursement for visit	1.8 (1.2)	1.6 (1.0)
No-show rate is higher	4 (0.8)	4.2 (0.7)
Families do not believe their child has OW/OB	3.5 (0.7)	3.8 (0.8)
Families choose not to schedule visits for OW/OB	3.7 (0.5)	3.8 (0.7)
Your belief visits are not worthwhile	2.3 (1.5)	1.6 (0.9)

Post-intervention parent survey responses indicated that parents generally felt pediatricians listened, valued their input, supported habit changes, and were encouraging (Table 4).

**Table 4:** Effectiveness – Post Intervention Parent Report of Patient-Centered Care by Pediatric Provider.

Questions	n = 15, n (%)
My pediatrician listened to me	
Strongly agree	7 (46.7)
Agree	6 (40.0)
Undecided	1 (6.7)
Disagree	0 (0.0)
Strongly disagree	1 (6.7)
My pediatrician asked my opinion	
Strongly agree	5 (33.3)
Agree	10 (66.7)
Undecided	0 (0.0)
Disagree	0 (0.0)
Strongly disagree	0 (0.0)
My pediatrician helped me change habits	
Strongly agree	6 (40.0)
Agree	9 (60.0)
Undecided	0 (0.0)
Disagree	0 (0.0)
Strongly disagree	0 (0.0)
My pediatrician is supportive and encouraging	
Strongly agree	6 (40.0)
Agree	8 (53.3)
Undecided	1 (6.7)
Disagree	0 (0.0)
Strongly disagree	0 (0.0)

*Note*: 18 of the 33 participants who completed the follow-up survey did not complete the follow-up survey in full, leaving questions unanswered.

Overall, parents reported observing healthy changes across the entire family unit. Families noted “drastic change[s]”, ranging from “significant weight loss” and reduced screen time to improvements in their child’s depression. Families who had previously been “barely walking” were now taking thousands of steps every day. One parent shared, “It works wonders if you actually learn to like it [healthy habits].” Even a participant who completed only part of the online program commented, “The small part [of DK!] I was able to experience helped me think more about it, so I put it into practice whenever I have the chance… the few things I saw helped me remember and apply it.”

Across all three educational foci—reducing the consumption of sugar-sweetened beverages, increasing physical activity, and following MyPlate recommendations—engaged parents shared success stories. Some self-reported behaviors (measured posttest only) were individual (parent-centric), while others reflected shared family decisions. For example, one parent reported, “I was used to buying them, you know, like Capri Suns or Kool-Aid – the pouches – and I took them away,” while another parent shared that “We chose to walk [to and from school].” When reflecting on dietary changes, one parent noted, “It was really challenging, and a great change for the three of us” (S5 Appendix).

In provider focus groups, participants reflected that “it’s helpful to have another tool” that serves as “a constant reminder to help with motivation” during patient visits. Given that providers typically have “15 minutes, 20 tops, maybe,” with each patient, they noted that time constrains limit their ability to address all aspects of weight management. As one provider explained, “We don’t get to talk about all things… regarding weight managing that we [they] would like to…the good thing was that, for ourselves, we took extra time with our patients.” Overall, providers reported that the program “worked very well” for motivated families. However, one provider noted mixed results, stating, “I do see the good result on 50 percent, and 50 percent didn’t work.”

### Adoption

Out of the 100 families who consented, 73 completed the baseline survey, and 46 engaged with the DK! website [17,28]. Additionally, while families anticipated spending several hours on the website, actual usage data showed that families spent an average of 144 minutes (SD = 132) on the DK! website over an average period of 89 days (SD = 77), with a range of one to 262 days. Six of the 46 families used the DK! goal-setting and tracking feature, collectively achieving 34 out of 52 possible goal days (65%). Over a third of families (38%) attended a planned follow-up visit, including 21 website users (46%) and seven non-users (26%) (Table 5).

**Table 5:** DK! Utilization.

Characteristics	n = 46
Average number of days per module on DK!	
Days on Sugar-Sweetened Beverages Module, Mean (SD)	10.2 (17.4)
Days on Physical Activity [as above], Mean (SD)	3.6 (11.0)
Days on MyPlate, Mean (SD)	8.9 (38.0)
Number of Days between first and last access	
Mean (SD)	89.3 (75.6)
Range [minutes]	1-262
Quartile 1	20.4
Quartile 2	72.3
Quartile 3	137.9
Quartile 4	261.5
Usage Duration	
Users who used less than 3 months, n (%)	23 (50.0)
Users who used more than 3 months, n (%)	23 (50.0)
Total amount of time spent in DK! website	
Mean (SD) minutes (Range: 4–476)	144.4 (132.1)
Goal-setting feature	
Number of users, n (%)	6 (13.0)
Number of goal days set, n	52
Number of goal days achieved, n (%)	34 (65.4%)

Fifteen of the 46 families provided further insights into why parents engaged with the program through the post-intervention survey. Fourteen (93%) reported that DK! influenced positive changes in both their child’s and their own health behaviors. Additionally, 12 of the 15 families (80%) reported sharing information they learned from DK! with others (Table 6).

**Table 6:** Patient-level adoption outcomes from the post intervention parent survey.

Questions:	n (%)
How important to you personally was the information from DK!?	n = 15
Completely	10 (66.7)
Very much so	4 (26.7)
Some	1 (6.7)
Did the information shared by DK! cause you to change any of your health behaviors?	n = 15
Yes	14 (93.3)
No	1 (6.7)
Did the information shared by DK! cause you to change any of your child’s health behaviors?	n = 15
Yes	14 (93.3)
No	1 (6.7)
Did you share any of the information you received from DK! with any of your friends or other family members?	n = 13
Yes	12 (92.3)
No	1 (7.712)

*Note*: 18 of the 33 participants who completed the follow-up survey did not complete the follow-up survey in full, leaving sections of questions unanswered.

The healthcare system developed and implemented the DK! tool within the EHR. All ten providers across the three primary care clinics agreed to participate and successfully integrated DK! into care by offering it at least once. Specifically, these providers offered the intervention to at least 256 of the 581 (44%) eligible patients, with stratified results published previously [28].

During the post-intervention interviews, families shared positive feedback about the program. One parent stated, “I liked all of it…All of it was beneficial,” while another noted, “we liked the whole program, you can learn beyond what you are expecting…as a mom, is that I feel very happy to have been able to receive the program.” Some parents reported that their children read all the information on the website and took the lead in participating, while others shared that they, the parents, “read everything.” Some families reported spending 30 minutes on the site daily, while others took two days to cover each topic. One parent reflected that they lost track of time “because he [my son] was involved with me, and we’d spend time doing it together.” Parents also acknowledged that one of the biggest challenge in adopting the program was confronting their own habits: “We realize that it’s something that we’re not doing daily. I think that’s the part I didn’t enjoy.”

Providers reflected that “parents were very interested” and many “seemed to be motivated.” They shared their perceptions of which families were best suited for the program. One provider reflected that DK! was more appropriate for “bigger [older] kids who can be motivated and do follow-up themselves,” whereas another provider believed that it was better suited for patients who “have had a rapid increase in weight gain.” In some cases, providers’ perceptions influenced whom they recruited to the program. One provider reported, “I didn’t even offer” unmotivated patients DK! because “I knew they’re not going to do it.” Providers expressed appreciation for the program’s personalized nature, noting that it was “more interactive for the kids…than a regular nutrition referral,” and that it was accessible online. While the format “gives parents the freedom…to have access away from a kind of formalized medical setting,” the intervention itself “empowered parents to take on more responsibility in addressing and facing their child’s issues around weight.” Overall, providers reflected that “for patients who were committed to it or interested in it, they felt that it was easy to use, and they actually enjoyed it.”

### Implementation

Patients accessed DK! in various settings and at different times. Of the 13 respondents, ten (77%) reported using the program in the afternoon or evening, while three participants (23%) used DK! in the morning. Among the 15 respondents, 14 (93%) families accessed DK! at home, whereas one family (7%) reported accessing it at church. Of the 14 respondents who answered the device-use question, eight (57%) reported using DK! on their computer, four (29%) on their smartphone, and two (14%) on both devices. All respondents felt that the number of topics and reminders was appropriate. Over half (57%) of families chose the sugar-sweetened beverage module as their first topic. 14 of the 15 parents (93%) reported that they “very much” or “completely” trusted the information and found it personally important. 13 of the 15 parents (87%) felt the content “very much” or “completely” addressed their needs and applied to their lives (Table 7).

**Table 7:** Implementation – Family Experience with the DK! Website, Aggregated from the DK Website and Guardian Surveys.

Questions	n (%)
Where did families typically review the information from Dynamo Kids! *(from question asked verbally of 15 participants in post intervention interview)*	n = 15
At home	14 (93.3)
At church	1 (6.7)
On what platform did users predominately use DK! *(from post-intervention survey)*	n = 15
Computer only	8 (53.3)
Cellphone only	4 (26.7)
Both	2 (13.3)
Initial module selected by user *(from Dynamo Kids! use data)*	n = 46
Sugar-sweetened beverages	26 (56.5)
Physical activity	10 (21.7)
MyPlate	10 (21.7)
How much did users feel the information from DK! “focused especially on you and your needs” *(from post-intervention survey)*	n = 15
Completely	8 (53.3)
Very much so	5 (15.15)
A little	2 (6.06)
How much did you feel the information from Dynamo Kids! applied to your life *(from post-intervention survey)*	n = 15
Completely	6 (40.0)
Very much So	7 (46.7)
Some	1 (6.7)
A little	1 (6.7)
How much did you trust the information from Dynamo Kids! to be accurate *(from post-intervention survey)*	n = 15
Completely	6 (40.0)
Very much So	8 (53.3)
Some	1 (6.7)
Do you feel that the number of topics included in Dynamo Kids was *(from post-intervention survey)*	n = 15
Just right	15 (100.0)
Do you feel that the number of reminders by text/email was *(from post-intervention survey)*	n = 15
Just right	15 (100.0)

*Note*: At least 18 of the 33 participants who completed the follow-up survey did not complete the follow-up survey in full, leaving sections of questions unanswered.

End-of-module feedback from the DK! website showed 97% of responses indicated that the information was easy to understand, relevant, and presented within an appropriate timeframe. Additionally, 99% of responses stated the information was clearly organized. Despite the program’s low overall uptake, 97% of respondents found the tracking tool helpful. Just over half of the responses (54%) indicated that cost was not a barrier to participating in activities. In summary, although not all participants used the DK! website, user feedback suggested the website was informative, trustworthy, and user-friendly (Table 8).

**Table 8:** Implementation – family experience with DK! website data aggregated from within the dk website, asked at the end of each module.

Questions	Response: Agree (A little or a lot) n (%)
I was able to understand the information	84 (96.6)
The challenge activities helped me understand the content	82 (94.3)
The colors and layout were pleasant to use	84 (96.6)
The amount of time I spent was just right	85 (97.7)
The tracking activities were helpful for my child and me	84 (96.6)
The doctor answers were helpful for my family and me	85 (97.7)
The family stories were role models for my family	82 (94.3)
The information was arranged in a clear way	86 (98.9)
The information was relevant to me and my child	84 (96.6)
Finances or costs served as a barrier [to making a lifestyle change]	49 (46.2)
**Questions**	**Response Selected:** **n (%)**
Who was the most important social support?	
Spouse or partner	65 (61.3)
Grandparents	7 (6.6)
Other family members	17 (16.0)
Doctor or health care provider	7 (6.6)
Child’s care giver	4 (3.8)
No one	6 (5.7)

*Notes*: Likert Scale options were Agree a lot, Agree a little, Disagree a little, and Disagree a lot. These questions were asked to each participant three times, once at the end of each module. The sample size, or count, is the total number of responses to this question throughout the entire intervention. In other words, assuming the user had completed the full intervention, the total possible count for each answer would be 138, as each user (n = 46) could answer this question at the end of each of the three modules. However, not every user completed the intervention in full.

On a systems level, the intervention largely adhered to the *a priori* study protocol. The BPA alert in the EHR was successfully developed, implemented, and correctly identified eligible patients. Providers received the BPA and offered the intervention to patients in most, though not all, cases. One of nine providers (11%) reported challenges sending electronic referrals through the BPA alert to enroll patients. Intervention fidelity was also assessed at follow-up visits using a provider-completed fidelity checklist, which was collected for 16 of 28 visits (57%). No significant IT issues were reported by families, apart from minor password reset requests. However, research assistants deviated from the original evaluation plan and did not complete follow-up data collection for all families, resulting in evaluation gaps.

Families reported that DK! was “so user-friendly” and that they learned a lot “because the information was so easy to understand.” They “liked the fact that it was online…. and we [families] didn’t have to go anywhere.” Families also shared positive feedback about the frequent parent-child interactions and felt a connection to the family stories “that let me know that there were real people dealing with similar issues to what I was dealing with.” They shared that they enjoyed the recipes and found the resource section was very helpful—one parent mentioned that they found local parks they “didn’t even know existed and were that close [to their home].”

One of the commonly cited barriers to participation included the weather, as rain and heat prevented families from engaging in outdoor physical activities. Some parents shared that certain information “was too real” and that “they didn’t want to hear” it. One parent reflected that, due to time constraints, “we [my child and I] rarely had time to sit down and go through the program together.” Parents also requested more illustrations and audio features, as well as additional content on parenting, sleep, and rice-based recipes, especially in the Spanish version for Spanish-speaking families. While families found the technology easy to use and navigate, their biggest frustration was having to wait for the next topic to become available after completing each module.

In general, providers found the BPA alert helpful and appreciated that “DK! was bilingual.” They affirmed that offering DK! did not slow down the visit, and it was “a really easy process to do the referral.” Another provider shared she “liked that it provided balance [in terms of content].” Providers also found the customized follow-up visit summary sheet, used alongside the follow-up visit checklist, helpful in orienting them to what to expect during the visit because this resource enriched the follow-up discussions, allowing providers to address more than just the child’s %BMI_p95_. Additionally, most providers agreed that the program’s online format—especially during the COVID-19 pandemic—was critical. However, one provider shared that she preferred paper documents over electronic ones and was hesitant to change the way she asked questions during visits. In her own words, “I am old, and I’m kind of set in my ways,” suggesting that additional support may be necessary for providers, who are less comfortable with technology.

Providers also suggested that DK! could be better integrated into the healthcare system by coordinating with nurses, social workers/health educators, and nutritionists. For example, one provider recommended scheduling a follow-up appointment with a nutritionist after patients engage with DK!, while another suggested having a full-time patient educator available in the clinic to help onboard and orient patients to the DK! website. Importantly, providers also expressed interest in implementing an additional layer of follow-up for patients who enrolled in DK! but did not actively engage with the program.

### Assessing selection bias

When interviewing parents who used DK!, they reported that their biggest challenges were competing priorities and time constraints. One parent, who had recently separated from her husband and had to move, shared that if she were to use DK! in the future, it “may bring me [her] and my [her] son together.” Another parent described personal changes that led her to move in with her sister-in-law, where the poor internet connectivity made it difficult to engage with the program.

From the provider perspective, potential barriers for patients who enrolled but did not use DK! included a patient population that is “not very computer-oriented,” the family’s “lack of motivation,” or difficulty translating the information into practice.

### Maintenance

In this DK! pilot program, there was adequate staffing and infrastructure to deliver DK!, resulting in both statistically and clinically significant outcomes [17,28]. Currently, DK! is being offered at a different clinic and evaluated as a streamlined referral service rather than a research study, and former participants continue to have access to the DK! website for ongoing engagement.

Both providers and families have expressed strong interest in extending the DK! program beyond the pilot phase. Some patients have inquired about new content creation, stating, “since I’m out of the program, is there a way I can … look at different stuff?” Providers have echoed this enthusiasm, with many expressing that “there’s ways that it can be expanded” and asking if they could continue offering DK!. They expressed willingness to do “whatever we can do to help” to continue offering DK! because “it’s a really good resource.” Program designers are actively seeking additional funding to explore opportunities for scaling and sustaining DK! long-term.

## Discussion

This pilot study used the RE-AIM framework to evaluate the DK! intervention across three sites within a safety-net health system in Dallas, TX. Grounded in the OCCM and guided by evidence-based strategies, this innovative eHealth intervention was successfully developed and implemented in three primary care clinics within a safety-net hospital. Designed for children ages 6–12 with OW/OB during the COVID-19 pandemic, the DK! program equitably targeted high-risk populations and demonstrated effectiveness by enhancing parent-reported family nutrition and physical activity behaviors, and by reducing children’s %BMI_p95_ as previously reported [46]. Of the 581 eligible patients, 215 were offered the intervention, 73 enrolled, and 46 patients completed the DK! intervention. Both participants and providers found that the intervention to be user-friendly and engaging. Additionally, stakeholders expressed a strong interest in continuing to offer and engage with DK! in the future. Significantly, by supporting high-risk populations with limited access to care, DK prioritized global health equity [51,52] using cultural, geographic, and linguistic tailoring.

In terms of intervention structure, parents appreciated the self-paced nature of the program, the breadth of information provided, and the ease of understanding the content. However, families engaged with the DK! website for significantly less time than anticipated based on formative research [35,53]. They suggested that future versions of the program include audio narration of the text so that they would not have to read as much. Additionally, families recommended removing the built-in time delay between modules, allowing them to move on to the next topic without having to wait a week.

Family feedback highlighted the importance of offering a broad variety of content to address the interests of different cultures and preferences. Specifically, families requested a sleep hygiene module, more culturally aligned recipes, and additional parenting advice. First, they expressed a desire for a module or more information on the importance of sleep and healthy sleep hygiene. Research has shown that sleep plays a crucial role in pediatric weight management, with increased sleep contributing to reduced excess caloric consumption [54]. Second, family feedback identified that rice is more prominent in Latino diets and suggested that additional time should be spent discussing this common food within the program, including more recipes with rice [55]. Finally, parents requested more guidance on parenting, recognizing the significant role parents play in shaping their child’s environment, behaviors, and diet [56]. This request aligns with findings from other pediatric weight management interventions that emphasize the importance of parental involvement. Future work could expand the topic options and ensure the content is culturally adapted to the target patient population.

Our findings align with and extend the limited body of research on eHealth [18] interventions for pediatric weight management. For example, while our eHealth intervention demonstrated significant improvements similar to those reported in the 2024 Greenlight Plus randomized clinical trial, their focus was on infants (0–2 years of age), whereas our study targeted children aged 6–12 years [57]. A second recent trial, the 2023 Aim2Be randomized controlled study, examined adolescents aged 10–17 years, a population with some overlap with ours, but assessed outcomes only at three months rather than at one year, as in DK! Importantly, unlike our study, that intervention did not demonstrate significant improvements in clinical outcomes.[58] Finally, a 2024 Italian study targeting the same age range as ours found that eHealth modalities promoted longer engagement, although they did not yield significant improvements in clinical measures [59].

While most systematic reviews on pediatric weight management interventions have focused exclusively on clinical trials [49,60], this study was developed in accordance with findings from a more recent systematic review emphasizing the importance of rigorously evaluating implementation science outcomes in studies beyond randomized trials as well [31]. Taken together, these comparisons highlight that Dynamo Kids! contributes important effectiveness and implementation science evidence demonstrating that family-based eHealth interventions can be both effective in producing meaningful, clinically relevant improvements in children ages 6–12 and responsive to calls for broader evaluation frameworks that capture real-world implementation outcomes.

### Strengths

First, our study used a mixed-methods approach that triangulated both quantitative and qualitative data from enrolled participants, including both users and non-users, as well as EHR data from eligible patients and providers. Second, while DK! was not initially developed to address the COVID-19 pandemic, its launch coincidentally aligned with the onset of the pandemic. This timing and delivery mechanism enabled DK! to serve as a timely, well-planned, and evidence-based solution to meet a real-world need. Third, guided by health equity principles and aligned with the OCCM, DK! was integrated into the safety-net healthcare system. This integration allowed for a comprehensive approach, with diagnoses and physiological measurements conducted within a trusted clinical setting, while the intervention itself remained home-based and accessible online.

### Limitations

A main limitation of our study was the small sample size of participants completing post-test surveys and engaging with the website. Several factors may explain the high attrition rate. In general, clinical weight management programs often report low participation and high dropout rates [61]. In this study, the pre- and post-test surveys were lengthy (100 + questions). While these surveys captured important data that helped us assess both our *a priori* effectiveness and implementation science outcomes and participants were financially compensated for their time, we hypothesized the length of the surveys contributed to a significant loss to follow up [62,63]. Moreover, the small sample size of this pilot study limited our ability to conduct subgroup analyses to explore intervention dosage or participant characteristics that may influence engagement and outcomes in future studies. Although our sample included a diverse population in terms of race/ethnicity, insurance coverage, and language preferences, the small scale and specific clinical setting may limit generalizability to other healthcare environments. Finally, the lack of control group restricts our ability to determine the comparative impact of the intervention. Given the concurrent COVID-19 pandemic, it is possible that a control group may have experienced a greater-than-typical increase in BMI, complicating interpretation of intervention effects [61,64].

### Future research

Given that this pilot study has inherent limitations in scale and generalizability, future research should focus on implementing DK! with a larger sample size to enable subgroup analyses and inclusion of a control group, ideally conducted outside the context of the COVID-19 era. It should also explore the development of culturally tailored versions of DK! for other at-risk populations and in different healthcare settings. Finally, given that existing recommendations for family-based behavioral interventions in pediatric weight management are largely based on pre-pandemic, in-person clinical trials, best practices for behavioral eHealth interventions remain unclear [18]. With the rapid adoption of eHealth approaches in recent years, rigorous evaluation of outcomes across in-person, hybrid, and remote settings is essential [18,65].

## Conclusion

Rates of OW/OB and their associated adverse outcomes, are increasing globally and inequitably, exacerbating disparities in health and health outcomes. Due to the complex, multifactorial nature of obesity, a single solution is insufficient for addressing the needs of all populations. Instead, we need to develop sustainable and scalable interventions that can be tailored to the unique needs of diverse users. The community stakeholder-informed, evidence-based DK! program successfully engaged a publicly insured, predominantly Spanish-speaking population within a safety-net health system in the southern United States. DK! led to improvements in both clinical and subjective patient-reported outcomes, with strong endorsements from both families and providers who supported the program’s features and its expansion. Future research will focus on scaling and adapting the program for additional at-risk populations, guided by the RE-AIM framework to ensure reach, effectiveness, adoption, implementation, and maintenance.

## Supporting information

S1 Appendix English Baseline Survey Qualtrics. Pre Survey.
(DOCX)

S2 Appendix English Post Survey. Post Survey.
(DOCX)

S3 Appendix PCP survey. Primary Care Provider Survey.
(DOCX)

S4 Appendix DK Process checklist_PCP. Dynamo Kids! Patient Discussion Summary.
(DOCX)

S5 Appendix Posttest Questions Table, Post Survey on DK! Website.
(DOCX)
