# Supplementary material for: Implementation science evaluation of an eHealth pediatric primary-care overweight and obesity intervention using the RE-AIM evaluation framework
Source: PLoS One. 2026 Feb 9;21(2):e0341635. doi: 10.1371/journal.pone.0341635 (PMC12885277; doi:10.1371/journal.pone.0341635)
Supplement: S1 Appendix — Pre Survey. (DOCX) [file pone.0341635.s001.docx]

How old are you?

- < 25 years
- 25-34
- 35-44
- 45-54
- 55-64
- ≥ 65

What is your gender?

- Male
- Female

Do you work for pay?

- No
- Yes, part time
- Yes, full time

You joined this program because you are the main caregiver of a child aged 6 to 12 years who is seen at this pediatric practice. Please check the relationship you have with that child.

- Parent
- Grandparent
- Other

Which of the following best describes your race/ethnicity? (Check all that apply)

- Black or African American
- Hispanic
- White or Caucasian
- Asian
- Other

What was your highest level of school?

- Some high school or less
- High school degree or GED
- College classes (no degree)
- College degree
- Additional education past college

What is the total yearly income in your household? Include income from all people working in the household and any child support payments.

- < $10,000
- $10,000-$19,999
- $20,000-$29,999
- $30,000-$39,999
- $40,000-$49,999
- $50,000-$59,999
- $60,000-$69,999
- ≥ $70,000

Please describe your access to a car.

- No car available
- Car available to me sometimes
- Car available to me most of the time

Please describe your home access to the internet.

- I do not get to the internet
- Cell phone access only
- Cell and home internet (Wi-Fi) access

How much do you currently weigh?

- In Pounds (lbs):

How tall are you?

- ____________ feet and ______________ inches

Choose only one: Are you currently…

- Married
- Widowed
- Living with a partner
- Single, never married
- Divorced/Separated

Please list all the other people (by their relationship, not name) who live in your household and their ages.

Example 1: Son, 7 Years

Example 2: Mother, 54 Years

- Click to write Choice 1
- Click to write Choice 2
- Click to write Choice 3
- Click to write Choice 4
- Click to write Choice 5
- Click to write Choice 6
- Click to write Choice 7
- Click to write Choice 8
- If more people live in your household, please write in here.

What do you think about the weight of your child?

- Underweight
- About the right weight
- Slightly overweight
- Very overweight

Please indicate if a physician has diagnosed you with any of the following conditions:

- High Blood Pressure
- High Cholesterol
- Heart Disease
- Stroke
- Liver disease
- Cancer Type:
- Depression
- Other Psychological Disorder:
- Eating Disorder Type
- Type 1 Diabetes
- Type 2 Diabetes
- Drug or alcohol addiction
- Weight loss surgery

Please indicate if a physician has diagnosed a member for your child’s family (child’s parent or child’s grandparent) with any of the following conditions:

- High Blood Pressure
- High Cholesterol
- Heart Disease
- Stroke
- Liver disease
- Cancer Type:
- Depression
- Other Psychological Disorder:
- Eating Disorder Type:
- Type 1 Diabetes
- Type 2 Diabetes
- Drug or alcohol addiction
- Weight loss surgery

Instructions: For each of the following questions, select the answer category that best fits your child or your family. It is important to indicate the most common or typical pattern for your family, and not what you would like to happen.

How often does your child eat breakfast, either at home or at school?

- Never/Almost Never
- Sometimes
- Often
- Very Often/Always

How often does your child eat at least one meal a day with at least one other family member?

- Never/Almost Never
- Sometimes
- Often
- Very Often/Always

How often does your child eat while watching TV? [Includes meals or snacks]

- Never/Almost Never
- Sometimes
- Often
- Very Often/Always

How often does your family eat “fast food?”

- Never/Almost Never
- Sometimes
- Often
- Very Often/Always

How often does your family use packaged “ready-‐to-‐eat” foods? [Includes purchased frozen or on-‐the-‐shelf entrees, often designed to be microwaved]

- Never/Almost Never
- Sometimes
- Often
- Very Often/Always

How often does your child eat fruits and vegetables at meals or snacks? [Not including juice]

- Never/Almost Never
- Sometimes
- Often
- Very Often/Always

How often does your child drink soda pop or sweetened beverages? [Includes regular or diet soda pop, Kool-‐Aid, Sunny-‐D, Capri Sun, sweet tea, fruit or vegetable juice, caffeinated energy drinks (Monster/Red Bull), Powerade/Gatorade, etc.]

- Never/Almost Never
- Sometimes
- Often
- Very Often/Always

How often does your child drink low-‐fat milk for meals or snacks? [Includes 1% or skim dairy, flavored, soy, almond, etc.]

- Never/Almost Never
- Sometimes
- Often
- Very Often/Always

How often does your family monitor the amount of candy, chips, and cookies your child eats?

- Never/Almost Never
- Sometimes
- Often
- Very Often/Always

How often does your family use candy, ice cream or other foods as a reward for good behavior?

- Never/Almost Never
- Sometimes
- Often
- Very Often/Always

How often does your child have less than 2 hours of “screen time” in a day? [Includes TV, computer, game system, or any mobile device with visual screens]

- Never/Almost Never
- Sometimes
- Often
- Very Often/Always

How often does your family monitor the amount of “screen time” your child has?

- Never/Almost Never
- Sometimes
- Often
- Very Often/Always

How often does your child engage in screen time in his/her bedroom?

- Never/Almost Never
- Sometimes
- Often
- Very Often/Always

How often does your family provide opportunities for physical activity?

- Never/Almost Never
- Sometimes
- Often
- Very Often/Always

How often does your family encourage your child to be physically active?

- Never/Almost Never
- Sometimes
- Often
- Very Often/Always

How often does your child do physical activities with at least one other family member?

- Never/Almost Never
- Sometimes
- Often
- Very Often/Always

How often does your child do something physically active when he/she has free time?

- Never/Almost Never
- Sometimes
- Often
- Very Often/Always

How often does your child participate in organized sports or physical activities with a coach or leader?

- Never/Almost Never
- Sometimes
- Often
- Very Often/Always

How often does your child follow a regular routine for your child’s bedtime?

- Never/Almost Never
- Sometimes
- Often
- Very Often/Always

How often does your child get enough sleep at night?

- Never/Almost Never
- Sometimes
- Often
- Very Often/Always

Sugar Sweetened Beverages: Knowledge

Select ALL drinks that count as a sugar-sweetened beverage.

- Coca Cola or Pepsi
- Sprite or 7-Up
- Gatorade
- Powerade
- Koolaid
- Sweet Iced Tea
- Unsweet Iced Tea
- White Milk
- Juice
- Chocolate Milk
- Water
- Sunny D or Capri Sun
- Ice Teas (Arizona teas or powdered teas)
- Energy Drinks like Monster and Redbull
- Lemonade

Sugar Sweetened Beverages: Attitude

How much do you agree or disagree with the following:

Sugary beverages are part of an active lifestyle

- Strongly Disagree
- Disagree
- Neutral
- Agree
- Strongly Agree

How much do you agree or disagree with the following:

It is okay to drink sugary drinks while pregnant

- Strongly Disagree
- Disagree
- Neutral
- Agree
- Strongly Agree

How much do you agree or disagree with the following:

Sugary drink consumption can negatively affect my child’s health

- Strongly Disagree
- Disagree
- Neutral
- Agree
- Strongly Agree

How much do you agree or disagree with the following:

Drinking sugary drinks increases the risk of gaining too much weight

- Strongly Disagree
- Disagree
- Neutral
- Agree
- Strongly Agree

Stage of Change:

For best health and to prevent too much weight, children should have no sugar-sweetened beverages (including 100% fruit juice) on most days.

Which of the following describes my child:

- Most days my child drinks no sugar-sweetened beverages (including 100% fruit juice)
- Most days my child drinks sugar-sweetened beverages

Are you thinking about ways to help your child get no sugar-sweetened beverages (including 100% fruit juice) on most days?

- Yes
- No

Are you definitely planning to help your child get no sugar-sweetened beverages (including 100% fruit juice) on most days?

- Yes
- No

Self-Efficacy: How sure are you that you can make certain your child gets no sugar-sweetened beverages (including 100% fruit juice) on most days?

- Very sure
- Sure
- Somewhat sure
- Somewhat unsure
- Unsure
- Very Unsure

Knowledge: For best health and to help children avoid gaining too much weight children should have physical activity over the day that adds up to:

- 10 minutes
- 30 minutes
- 45 minutes
- 50 minutes
- 60 minutes or more

Select ALL activities that count as physical activity

- Jumping Jacks
- Jogging
- Basketball
- Treadmill
- Playing Pokemon
- Reading a Book
- Dancing
- Playing Tag
- Hula Hoop
- Frisbee
- Walking to the store or to school
- Other

Attitudes:

How much do you agree or disagree with the following?

My exercise habits will strongly impact the exercise habits that my child will develop over the course of his/her life.

- Strongly Disagree
- Disagree
- Neutral
- Agree
- Strongly agree

How much do you agree or disagree with the following?

I am scared that physical activity will be harmful for my child.

- Strongly Disagree
- Disagree
- Neutral
- Agree
- Strongly agree

How much do you agree or disagree with the following?

My child will learn exercise habits through watching my example.

- Strongly Disagree
- Disagree
- Neutral
- Agree
- Strongly agree

How much do you agree or disagree with the following?

I worry that participating in physical activities or sports will be a bad experience for my child.

- Strongly Disagree
- Disagree
- Neutral
- Agree
- Strongly agree

How much do you agree or disagree with the following?

Activity improves functioning of my child’s cardiovascular system.

- Strongly Disagree
- Disagree
- Neutral
- Agree
- Strongly agree

How much do you agree or disagree with the following?

I am worried about my child’s ability to participate in sports or physical activities.

- Strongly Disagree
- Disagree
- Neutral
- Agree
- Strongly agree

How much do you agree or disagree with the following?

Physical activity increases my child’s mental alertness.

- Strongly Disagree
- Disagree
- Neutral
- Agree
- Strongly agree

How much do you agree or disagree with the following?

Increasing activity increases my child’s level of physical fitness.

- Strongly Disagree
- Disagree
- Neutral
- Agree
- Strongly agree

How much do you agree or disagree with the following?

Physical activity will make my child frustrated.

- Strongly Disagree
- Disagree
- Neutral
- Agree
- Strongly agree

How much do you agree or disagree with the following?

Physical activity increases my child’s muscle strength.

- Strongly Disagree
- Disagree
- Neutral
- Agree
- Strongly agree

How much do you agree or disagree with the following?

Exercising helps my child sleep better at night.

- Strongly Disagree
- Disagree
- Neutral
- Agree
- Strongly agree

How much do you agree or disagree with the following?

My child’s physical endurance is improved by encouraging him/her to be active.

- Strongly Disagree
- Disagree
- Neutral
- Agree
- Strongly agree

How much do you agree or disagree with the following?

Physical activity improves my child’s flexibility.

- Strongly Disagree
- Disagree
- Neutral
- Agree
- Strongly agree

How much do you agree or disagree with the following?

My attitudes about exercise will strongly impact my child’s attitude towards exercise over the course of his/her life.

- Strongly Disagree
- Disagree
- Neutral
- Agree
- Strongly agree

How much do you agree or disagree with the following?

My child is not able to participate in group physical activity or sports programs.

- Strongly Disagree
- Disagree
- Neutral
- Agree
- Strongly agree

How much do you agree or disagree with the following?

I am scared that physical activity will lead to disappointment for my child.

- Strongly Disagree
- Disagree
- Neutral
- Agree
- Strongly agree

How much do you agree or disagree with the following?

My child has improved feelings of well-being from physical activity.

- Strongly Disagree
- Disagree
- Neutral
- Agree
- Strongly agree

How much do you agree or disagree with the following?

Physical activity gives my child a sense of personal accomplishment.

- Strongly Disagree
- Disagree
- Neutral
- Agree
- Strongly agree

How much do you agree or disagree with the following?

I will improve future health by encouraging physical activity in my child.

- Strongly Disagree
- Disagree
- Neutral
- Agree
- Strongly agree

How much do you agree or disagree with the following?

Physical activity in childhood will make my child healthier.

- Strongly Disagree
- Disagree
- Neutral
- Agree
- Strongly agree

How much do you agree or disagree with the following?

My child will live longer if I encourage him/her to be an active child.

- Strongly Disagree
- Disagree
- Neutral
- Agree
- Strongly agree

How much do you agree or disagree with the following?

Physical activity is good entertainment for my child.

- Strongly Disagree
- Disagree
- Neutral
- Agree
- Strongly agree

How much do you agree or disagree with the following?

How much I value exercise will impact how active my child is.

- Strongly Disagree
- Disagree
- Neutral
- Agree
- Strongly agree

How much do you agree or disagree with the following?

Physical activity improves overall body functioning for my child.

- Strongly Disagree
- Disagree
- Neutral
- Agree
- Strongly agree

How much do you agree or disagree with the following?

I worry that my child will not be accepted by others if he/she participates in a group sport or activity program.

- Strongly Disagree
- Disagree
- Neutral
- Agree
- Strongly agree

Stage of Change: The recommended daily amount of physical activity for children is 60 minutes or more.

Which best describes my child:

- Most days my child gets the recommended amount of daily exercise
- b) Most days my child does not get the recommended amount of daily exercise

How long has your child been getting the recommended amount of physical activity?

- Less than 1 month
- 1-3 months
- 4-6 months
- More than 6 months

Are you thinking about ways to help your child get the recommended amount of physical activity?

- No
- Yes

Are you definitely planning to help your child get the recommended amount of physical activity on most days?

- No
- Yes

Self-Efficacy

How sure are you that you can make certain your child gets the recommended amount of physical activity daily?

- Very Sure
- Sure
- Somewhat Sure
- Somewhat unsure
- Unsure
- Very Unsure

Please answer questions about MyPlate as they relate to your child.

MyPlate recommendations say that a healthy evening meal contains how many food groups?

- 3
- 4
- 5
- 6
- 7

Which of the following is the most nutritious choice for fruit?

- Orange juice
- V-8 Juice
- Raisins
- Fruit Cup in Syrup
- Banana

What is the recommended serving size for milk at a meal?

- ½ cup
- ¼ cup
- 1 cup
- 1 ½ cup
- 2 cups

What food group does rice belong in?

- Fruits
- Meats/Proteins
- Vegetables
- Grains
- Dairy
- Fat/Oils

Which of the following foods is the most nutritious choice for vegetables?

- Frozen green beans cooked in the microwave
- Broccoli-rice-cheese casserole
- Lettuce, tomato, carrot, and crouton salad with 1 tablespoon of ranch dressing
- Baked potato with green onions and sour cream

Which of these is the recommended portion for fruit at a meal?

- 1 cup sliced banana
- 1 small apple
- 5 small grapes
- 2 cups blueberries

What is the healthiest way to prepare meat/protein?

- Roasted
- Fried
- Breaded and baked
- Sautéed in butter

Which food group does bacon belong in?

- Fruits
- Meats/proteins
- Vegetables
- Grains
- Dairy
- Fat/Oils

There are lots of different options for grains and starches. This question is about amount. Which of the following choices has the recommended serving size?

- 2 slices of bread
- 1 cup of rice
- 1 ½ cups pasta
- ½ cup of ready-to-eat cereal
- 1 corn tortilla

Which of the following foods is an example of whole grains?

- Steamed white rice
- Regular cheerios
- Flour tortillas
- Cinnamon raisin bagel

Which food group is missing from the following meal:

Spaghetti noodles with meat sauce, Salad, An orange, Water to drink

- Protein/meat
- Dairy
- Fruit
- Grain/starches

What is the recommended portion for grains/starches at a meal?

- 1/4 cup cooked rice
- 2 cups pasta
- 1 slice bread
- 2 dinner rolls
- Which milk does MyPlate recommend?
- Unflavored whole milk
- Fat-free chocolate milk
- Unflavored 2% milk
- Unflavored fat free or 1% milk

Mark ALL the specific food groups that should be included on your / your child’s dinner plate for a complete MyPlate meal.

- Fruits
- Meats/proteins
- Vegetables
- Grains/starches
- Dairy
- Fat/Oils
- Dessert

Which is the recommended serving size for chicken?


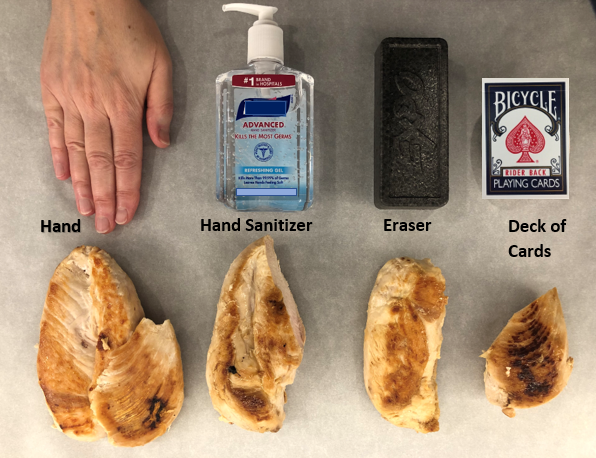


(This size is recommended for a serving of meat or fish as well.)

- About the size of an adult hand
- About the size of a bottle of hand sanitizer
- About the size of an eraser
- About the size of a deck of cards

Attitudes toward MyPlate:

How much do you agree or disagree with the following:

MyPlate is relevant to me

- Strongly Disagree
- Disagree
- Neutral
- Agree
- Strongly Agree

How much do you agree or disagree with the following:

MyPlate is easy to understand

- Strongly Disagree
- Disagree
- Neutral
- Agree
- Strongly Agree

How much do you agree or disagree with the following:

MyPlate will help my kids eat better

- Strongly Disagree
- Disagree
- Neutral
- Agree
- Strongly Agree
- MyPlate recommends that a healthy meal include fruit, grains, vegetables, protein, and dairy.

Which of the following describes your child having 3 MyPlate dinners each week?

- Most weeks my child has 3 MyPlate dinners per week
- Most weeks my child does not have 3 MyPlate dinners per week

How long has your child been having 3 MyPlate dinners per week?

- Less than 1 month
- 1-3 months
- 4-6 months
- More than 6 months
- Are you thinking about ways to help your child get 3 MyPlate dinners each week?
- No
- Yes
- Are you definitely planning to help your child get 3 MyPlate dinners each week?
- No
- Yes

Self-efficacy:

How sure are you that you can make 3 MyPlate meals each week

- Very Sure
- Sure
- Somewhat Sure
- Somewhat Unsure
- Unsure

Food Insecurity: please complete each statement for your household.

Within the past 12 months, we worried whether our food would run out before we got money to buy more.

- Often true
- Sometimes true
- Never true

Within the past 12 months, the food we bought just didn’t last and we didn’t have money to get more.

- Often true
- Sometimes true
- Never true
