# Supplementary material for: Implementation science evaluation of an eHealth pediatric primary-care overweight and obesity intervention using the RE-AIM evaluation framework
Source: PLoS One. 2026 Feb 9;21(2):e0341635. doi: 10.1371/journal.pone.0341635 (PMC12885277; doi:10.1371/journal.pone.0341635)
Supplement: S2 Appendix — Post Survey. (DOCX) [file pone.0341635.s005.docx]

1. What do you think about the weight of your child?

*Circle your opinion of their weight*.

| Underweight | About the right weight | Slightly overweight | Very overweight |
| --- | --- | --- | --- |

| **Instructions:** For each question, select the answer category that best fits your child or your family. It is important to indicate the most common or typical pattern for your family, and not what you would like to happen. | | | | |
| --- | --- | --- | --- | --- |
|  |  |  |  |  |
| **Family Meals** | Never/ Almost Never | Sometimes | Often | Very Often/ Always |
| 2.. How often does your child eat breakfast, either at home or at school? | 1 | 2 | 3 | 4 |
| 3.. How often does your child eat at least one meal a day with at least one other family member? | 1 | 2 | 3 | 4 |
| **Family Eating Practices** | Never/ Almost Never | Sometimes | Often | Very Often/ Always |
| 4.. How often does your child eat while watching TV? [Includes meals or snacks] | 1 | 2 | 3 | 4 |
| 5.. How often does your family eat “fast food?” | 1 | 2 | 3 | 4 |
| **Food Choices** | Never/ Almost Never | Sometimes | Often | Very Often/ Always |
| 6.. How often does your family use packaged “ready-­‐to-­‐eat” foods? [Includes purchased frozen or on-­‐the-­‐shelf entrees, often designed to be microwaved] | 1 | 2 | 3 | 4 |
| 7.. How often does your child eat fruits and vegetables at meals or snacks? [Not including juice] | 1 | 2 | 3 | 4 |
| **Beverage Choices** | Never/ Almost Never | Sometimes | Often | Very Often/ Always |
| 8.. How often does your child drink soda pop or sweetened beverages? [Includes regular or diet soda pop, Kool-­‐Aid, Sunny-­‐D, Capri Sun, sweet tea, fruit or vegetable juice, caffeinated energy drinks (Monster/Red Bull), Powerade/Gatorade, etc.] | 1 | 2 | 3 | 4 |
| 7.. How often does your child drink low-­‐fat milk for meals or snacks? [Includes 1% or skim dairy, flavored, soy, almond, etc.] | 1 | 2 | 3 | 4 |
| **Restriction/Reward** | Never/ Almost Never | Sometimes | Often | Very Often/ Always |
| 10.. How often does your family monitor the amount of candy, chips, and cookies your child eats? | 1 | 2 | 3 | 4 |
| 11.. How often does your family use candy, ice cream or other foods as a reward for good behavior? | 1 | 2 | 3 | 4 |
| **Screen Time** | Never/ Almost Never | Sometimes | Often | Very Often/ Always |
| 12. How often does your child have less than 2 hours of “screen time” in a day? [Includes TV, computer, game system, or any mobile device with visual screens] | 1 | 2 | 3 | 4 |
| 13. How often does your family monitor the amount of “screen time” your child has? | 1 | 2 | 3 | 4 |
| **Healthy Environment** | Never/ Almost Never | Sometimes | Often | Very Often/ Always |
| 14. How often does your child engage in screen time in his/her bedroom? | 1 | 2 | 3 | 4 |
| 15. How often does your family provide opportunities for physical activity? | 1 | 2 | 3 | 4 |
| **Family Activity** | Never/ Almost Never | Sometimes | Often | Very Often/ Always |
| 16. How often does your family encourage your child to be physically active? | 1 | 2 | 3 | 4 |
| 17. How often does your child do physical activities with at least one other family member? | 1 | 2 | 3 | 4 |
| **Child Activity** | Never/ Almost Never | Sometimes | Often | Very Often/ Always |
| 18. How often does your child do something physically active when he/she has free time? | 1 | 2 | 3 | 4 |
| 19. How often does your child participate in organized sports or physical activities with a coach or leader? | 1 | 2 | 3 | 4 |
| **Family Schedule/Sleep Routine** | Never/ Almost Never | Sometimes | Often | Very Often/ Always |
| 20. How often does your child follow a regular routine for your child’s bedtime? | 1 | 2 | 3 | 4 |
| 21. How often does your child get enough sleep at night? | 1 | 2 | 3 | 4 |

**Sugar Sweetened Beverages:**

**Knowledge**

1. Put a check next to each drink that counts as a sugar-sweetened beverage

Coca Cola or Pepsi

Sprite or 7-Up

Gatorade

Powerade

Koolaid

Sweet Iced Tea

Unsweet Iced Tea

White Milk

Juice

Chocolate Milk

Water

Sunny D or Capri Sun

Ice Teas (Arizona teas or powdered teas)

Energy Drinks like Monster and Redbull

Lemonade

**Attitudes**

How much do you agree or disagree with the following:

|  | Strongly Disagree | Disagree | Neutral | Agree | Strongly  Agree |
| --- | --- | --- | --- | --- | --- |
| 1. Sugary beverages are a part of an active lifestyle | 5 | 4 | 3 | 2 | 1 |
| 1. It is okay to drink sugary drinks while pregnant | 5 | 4 | 3 | 2 | 1 |
| 1. Sugary drink consumption can negatively affect my child’s health | 5 | 4 | 3 | 2 | 1 |
| 1. Drinking sugary drinks increases the risk of gaining too much weight | 5 | 4 | 3 | 2 | 1 |

**Stage of Change**

6a. For best health and to help children avoid gaining too much weight, children should have no sugar-sweetened beverages (including 100% fruit juice) on most days.

Which of the following describes my child:

1. Most days my child drinks no sugar-sweetened beverages (including 100% fruit juice)
2. Most days my child drinks sugar-sweetened beverages *(Skip to 6c)*

6b. How long has your child been drinking been drinking no-sugar sweetened beverages most days?

1. Less than 1 month
2. 1-3 months
3. 4-6 months
4. More than 6 months

| ***If answering 6b, skip to 7*** |
| --- |

6c. Are you thinking about ways to help your child get no sugar-sweetened beverages (including 100% fruit juice) on most days?

1. No
2. Yes

6d. If yes to 6c, are you **definitely planning** to help you child get no sugar-sweetened beverages (including 100% fruit juice) on most days?

1. No
2. Yes

**Self-efficacy**

1. How sure are you that you can make certain your child gets no sugar-sweetened beverages (including 100% fruit juice) on most days?
2. Very Sure
3. Sure
4. Somewhat sure
5. Somewhat unsure
6. Unsure
7. Very Unsure

**This section is about Physical Activity**

**Knowledge**

1. According to the Centers for Disease Control and Prevention (CDC), for best health and to help children avoid gaining too much weight children should have physical activity over the day that adds up to:

1. 10 minutes
2. 30 minutes
3. 45 minutes
4. 50 minutes
5. 60 minutes or more

2. Put a check next to all of the activities that count as physical activity

Jumping Jacks

Jogging

Basketball

Treadmill

Playing Pokemon

Reading a book

Dancing

Playing Tag

Hula Hoop

Frisbee

Walking to the store or to school

Other________________________

**Attitudes**

**Parent Perceptions about Physical Activity scale-preschool version**

How much do you agree or disagree with the following:

|  | Strongly Disagree | Disagree | Agree | Strongly  Agree |
| --- | --- | --- | --- | --- |
| 1. My exercise habits will strongly impact the exercise habits that my child will develop over the course of his/her life. | 4 | 3 | 2 | 1 |
| 1. I am scared that physical activity will be harmful for my child. | 4 | 3 | 2 | 1 |
| 1. My child will learn exercise habits through watching my example. | 4 | 3 | 2 | 1 |
| 1. I worry that participating in physical activities or sports will be a bad experience for my child. | 4 | 3 | 2 | 1 |
| 1. Activity improves functioning of my child’s cardiovascular system. | 4 | 3 | 2 | 1 |
| 1. I am worried about my child’s ability to participate in sports or physical activities. | 4 | 3 | 2 | 1 |
| 1. Physical activity increases my child’s mental alertness. | 4 | 3 | 2 | 1 |
| 1. Increasing activity increases my child’s level of physical fitness. | 4 | 3 | 2 | 1 |
| 1. Physical activity will make my child frustrated. | 4 | 3 | 2 | 1 |
| 1. Physical activity increases my child’s muscle strength. | 4 | 3 | 2 | 1 |
| 1. Exercising helps my child sleep better at night. | 4 | 3 | 2 | 1 |
| 1. My child’s physical endurance is improved by encouraging him/her to be active. | 4 | 3 | 2 | 1 |
| 1. Physical activity improves my child’s flexibility. | 4 | 3 | 2 | 1 |
| 1. My attitudes about exercise will strongly impact my child’s attitude towards exercise over the course of his/her life. | 4 | 3 | 2 | 1 |
| 1. My child is not able to participate in group physical activity or sports programs. | 4 | 3 | 2 | 1 |
| 1. I am scared that physical activity will lead to disappointment for my child. | 4 | 3 | 2 | 1 |
| 1. My child has improved feelings of well-being from physical activity. | 4 | 3 | 2 | 1 |
| 1. Physical activity gives my child a sense of personal accomplishment. | 4 | 3 | 2 | 1 |
| 1. I will improve future health by encouraging physical activity in my child. | 4 | 3 | 2 | 1 |
| 1. Physical activity in childhood will make my child healthier. | 4 | 3 | 2 | 1 |
| 1. My child will live longer if I encourage him/her to be an active child. | 4 | 3 | 2 | 1 |
| 1. Physical activity is good entertainment for my child. | 4 | 3 | 2 | 1 |
| 1. How much I value exercise will impact how active my child is. | 4 | 3 | 2 | 1 |
| 1. Physical activity improves overall body functioning for my child. | 4 | 3 | 2 | 1 |
| 1. I worry that my child will not be accepted by others if he/she participates in a group sport or activity program. | 4 | 3 | 2 | 1 |

**Stage of Change**

The recommended daily amount of physical activity for children is 60 minutes or more.

1. Which best describes my child:
2. Most days my child gets the recommended amount of daily exercise
3. Most days my child does not get the recommended amount of daily exercise *(Skips to 28c)*

28b. How long has your child been getting the recommended amount of physical activity?

1. Less than 1 month
2. 1-3 months
3. 4-6 months
4. More than 6 months

| ***If answering 28b, skip to 29*** |
| --- |

28c. Are you thinking about ways to help your child get the recommended amount of physical activity?

1. No
2. Yes

28d. If yes to 28c, are you **definitely planning** to help you child get the recommended amount of physical activity on most days?

1. No
2. Yes

**Self-Efficacy**

1. How sure are you that you can make certain your child gets the recommended amount of physical activity daily?
2. Very Sure
3. Sure
4. Somewhat sure
5. Somewhat unsure
6. Unsure
7. Very Unsure

**This section is about MyPlate**

The purpose of this questionnaire is to better understand what patients and parents know about MyPlate, the United States’ dietary guidelines for children. If you are a parent, please answer questions as they relate to your child’s (the patient) age.

| 1. Myplate recommendations say that a healthy evening meal contains how many food groups? | 3  4  5  6  7 |
| --- | --- |
| 1. Which of the following is the most nutritious choice for fruit? | Orange juice  V-8 juice  Raisins  Fruit cup in syrup  Banana |
| 1. What is the recommended serving size for milk at a meal? | ½ cup  ¼ cup  1 cup  1 ½ cup  2 cups |
| 4. What food group does rice belong in? | Fruits  Meats/proteins  Vegetables  Grains  Dairy  Fat/Oils |
| 5. Which of the following foods is the most nutritious choice for vegetables? | Frozen green beans cooked in the microwave  Broccoli-rice-cheese casserole  Lettuce, tomato, carrot, and crouton salad with 1 tablespoon of ranch dressing  Baked potato with green onions and sour cream |
| 6. Which of these is the recommended portion for fruit at a meal? | 1 cup sliced banana  1 small apple  5 grapes  2 cups blueberries |
| 7. What is the healthiest way to prepare meat/protein? | Roasted  Fried  Breaded and baked  Sautéed in butter |
| 8. Which food group does bacon belong in? | Fruits  Meats/proteins  Vegetables  Grains  Dairy  Fat/Oils |
| 9. There are lots of different options for grains and starches. Which of the following choices has the recommended serving size for a meal? | 2 slices of bread  2 cups rice  1 ½ cups pasta  ½ cup potato  1 corn tortilla |
| 10. Which of the following foods is an example of whole grains? | Steamed white rice  Regular cheerios  Flour tortillas  Cinnamon raisin bagel |
| 11. Which food group is missing from the following meal:   - Spaghetti noodles with meat sauce - salad - an orange - water to drink | Protein/meat  Dairy  Fruit  Grains/starches |
| 12. What is the recommended portion for grains/starches at a meal? | 1 cup cooked rice  2 cups pasta  1 slice bread  2 dinner rolls |
| 13. Which milk does MyPlate recommend? | Unflavored whole milk  Fat-free chocolate milk  Unflavored 2% milk  Unflavored fat free or 1% milk |
| 14. **Check ALL** the specific food groups that should be included on your / your child’s dinner plate for a complete MyPlate meal. | Fruits  Meats/proteins  Vegetables  Grains/starches  Dairy  Fat/Oils  Dessert |
| 15. What is the recommended serving size for cooked beans at dinner? | 1 cup  2 cups  1/2 cup  1 ½ cups |

**Attitudes toward MyPlate (from Wansink and Kranz 2013)**

How much do you agree or disagree with the following:

|  | Strongly Disagree | Disagree | Neutral | Agree | Strongly  Agree |
| --- | --- | --- | --- | --- | --- |
| 1. MyPlate is relevant to me | 5 | 4 | 3 | 2 | 1 |
| 1. MyPlate is easy to understand | 5 | 4 | 3 | 2 | 1 |
| 1. MyPlate will help my kids eat better | 5 | 4 | 3 | 2 | 1 |

MyPlate recommends that a healthy meal include fruit, grains, vegetables, protein, and dairy.

1. Which of the following describes your child having 3 MyPlate dinners each week?
2. Most weeks my child has 3 MyPlate dinners per week
3. Most weeks my child does not have 3 MyPlate dinners per week *(Skips to 19c)*

4b. How long has your child been having 3 MyPlate dinners per week?

1. Less than 1 month
2. 1-3 months
3. 4-6 months
4. More than 6 months

| ***If answering 4b, skip to 5*** |
| --- |

4c. Are you thinking about ways to help your child get 3 MyPlate dinners each week?

1. No
2. Yes

4d. If yes to 19c, are you **definitely planning** to help you child get 3 MyPlate dinners each week?

1. No
2. Yes

**Self-efficacy**

1. How sure are you that you can make **3 MyPlate meals** each week?
2. Very Sure
3. Sure
4. Somewhat sure
5. Somewhat unsure
6. Unsure
7. Very Unsure

**Food Insecurity**

1. Within the past 12 months, we worried whether our food would run out before we got money to buy more.
2. Yes
3. No
4. Within the past 12 months, the food we bought just didn’t last and we didn’t have money to get more.
5. Yes
6. No

**Process Evaluation Questions (for follow-up Survey only)**

1. Over the 3 months, do you recall getting any health-related lessons about child health on the internet or on your smartphone from Dynamo Kids!?
2. Yes
3. No
4. I don’t know

🡺 ***If "no" or "don't know", stop here.***

1. How many lessons or topics do you remember receiving?
2. 1
3. 2
4. 3
5. 4
6. I don’t know

3. What topics did the program cover? *[Mark all that apply]*

1. Physical activity
2. Sugar-sweetened beverages
3. MyPlate
4. Other ______________________
5. I don’t remember.

4. How much did you feel that the information from Dynamo Kids! focused especially on you and your needs?

1. Not at all
2. A little
3. Somewhat
4. Very much so
5. Completely
6. I don’t know
7. How important to you personally was the information from Dynamo Kids!?
8. Not at all
9. A little
10. Somewhat
11. Very much so
12. Completely
13. I don’t know
14. How much did the information from Dynamo Kids! apply to your life?
15. Not at all
16. A little
17. Somewhat
18. Very much so
19. Completely
20. I don’t know
21. Did the information shared by Dynamo Kids! cause you to change any of your health behaviors?
22. Yes
23. No
24. I don’t know

8. Did the information shared by Dynamo Kids! cause you to change any of your child’s health behaviors?

1. Yes
2. No
3. I don’t know

9. Did you share the information you received from Dynamo Kids! with any of your friends or family members?

1. Yes
2. No
3. I don’t know
4. How much did you trust the information from Dynamo Kids to be accurate?
5. Not at all
6. A little
7. Somewhat
8. Very much so
9. Completely
10. I don’t know
11. Do you feel that the number of topics included in Dynamo Kids! was:
12. Too few
13. Just right
14. Too many
15. I don’t know
16. How easy was it for you to access information from the internet?
17. Very easy
18. Somewhat easy
19. Not very easy
20. Very inconvenient
21. Where did you typically review the information from Dynamo Kids? *[Mark all that apply]*
22. At home
23. At work
24. At church
25. Other: _______________________
26. Do you feel that the number of reminders by text/email was:
27. Too few
28. Just right
29. Too many
30. I don’t know

|  | Disagree  a lot | Disagree a little | Undecided | Agree | Agree a lot |
| --- | --- | --- | --- | --- | --- |
| 1. My pediatrician listened to me regarding my child’s health issues | 5 | 4 | 3 | 2 | 1 |
| 1. My pediatrician asked my opinion regarding my child’s weight | 5 | 4 | 3 | 2 | 1 |
| 1. My pediatrician helped me think about changing my family’s food habits. | 5 | 4 | 3 | 2 | 1 |
| 1. My pediatrician helped me think about ways to improve/increase my child’s physical activity. | 5 | 4 | 3 | 2 | 1 |
| 1. My pediatrician was supportive and encouraging | 5 | 4 | 3 | 2 | 1 |

20. In the last 4 months, did you meet with a dietitian or nutritionist to discuss your child’s eating and nutrition? (Do not count any meetings about your eating and nutrition.)

a) No

b) One time

c)Two times

d) More than two times

21. In the last 4 months, did your child participate in any programs for the purpose of improving nutrition?

a) No

b) Yes

If yes, check all that apply

- Community-program
- School-run program
- Online/virtual program
- Other: (please give name______________________)

22. In the last 4 months, did your child participate in any programs for the purpose of improving physical activity?

a) No

b) Yes *(please give name _________________)*
