# Supplementary material for: Implementation science evaluation of an eHealth pediatric primary-care overweight and obesity intervention using the RE-AIM evaluation framework
Source: PLoS One. 2026 Feb 9;21(2):e0341635. doi: 10.1371/journal.pone.0341635 (PMC12885277; doi:10.1371/journal.pone.0341635)
Supplement: S3 Appendix — Primary Care Provider Survey. (DOCX) [file pone.0341635.s002.docx]

Primary Care Provider Survey

1. Please tell us what type of work you do in the practice:
   - I am an MD or DO
   - I am an NP or PA
   - I am a nurse
   - I am a medical assistant
   - Other, please specify
2. Years since medical training was completed years *(write in number)*
3. Gender
   - Female
   - Male
4. Race
   - Caucasian
   - African American
   - American Indian or Alaska Native
   - Asian
   - Pacific Islander/Native Hawaiian
   - Other
5. Ethnicity
   - Hispanic
   - Non-Hispanic
6. Do you communicate in Spanish (without need for a translator) with your Spanish-speaking patients?
   - Yes
   - No
7. In general, in this office, how far ahead are appointment templates created for scheduling?
   - 0-2 weeks
   - 3-4 weeks
   - 5-6 weeks
   - 2 mon
   - 3 mon
   - 4 mon
   - 5 mon
   - 6 or more mon
   - Don’t know
8. In general, in this office, when would an appointment be available (next available) for patients who want to schedule a visit?
   - 0-2 weeks
   - 3-4 weeks
   - 5-6 weeks
   - 2 mon
   - 3 mon
   - 4 mon
   - 5 mon
   - 6 or more mon
   - Don’t know
9. Do you have a visit template or structured note in your electronic health record that is specifically for a visit about overweight or obesity?
   - Yes
   - No
   - Don’t know
10. The CDC definition of overweight for children aged 6-12 years is
    - 91st -95th BMI
    - 95th-99th BMI
    - 85th-94th BMI
    - None of the above
11. The CDC definition of obesity for children 6-12 years is
    - >=94th BMI
    - >= 95th BMI
    - >=96th BMI
    - >=99th BMI
12. Among your patients who are between 6 and 12 years of age, approximately what percentage have BMIs in the overweight category on the BMI growth curve?
    - 5%
    - 10%
    - 15%
    - 20%
    - 25% or more
13. Among your patients who are between 6 and 12 years of age, approximately what percentage have BMIs in the obesity category on the BMI growth curve?
    - 5%
    - 10%
    - 15%
    - 20%
    - 25% or more
14. My practice has the following handouts for families related to childhood healthy lifestyle:

🞎

🞎

🞎

🞎

🞎

🞎

|  | Yes | No | Don’t  know |
| --- | --- | --- | --- |
| Community resources to support childhood healthy lifestyle (example  recreation programs, farmers markets) in English |  |  |  |
| Community resources to support childhood healthy lifestyle (example recreation programs, farmers markets) in Spanish |  |  |  |
| Tips for healthy lifestyle behaviors for children in English |  |  |  |
| Tips for healthy lifestyle behaviors for children in Spanish |  |  |  |

1. In the last 3 months, how many times did you have an encounter with a patient between 6 and 12 years of age in which the primary purpose was to address overweight or obesity? Do not include well child visits.

🞎

🞎

🞎

🞎

*(write in number)*

1. I or my staff routinely discuss BMI percentile with families of all pediatric patients with overweight
   - Yes
   - No
2. I or my staff routinely discuss BMI percentile with families of all pediatric patients with obesity
   - Yes
   - No

How much do you agree with the following statements when seeing a child with obesity/overweight?

🞎

🞎

🞎

🞎

🞎

🞎

🞎

🞎

🞎

🞎

🞎

| Strongly  Agree | | Agree | Neutral | Disagree | Strongly  Disagree |
| --- | --- | --- | --- | --- | --- |
| 18. Talking to a parent about a child’s  overweight or obesity is an uncomfortable discussion |  |  |  |  |  |
| 19. Parents often take offense when I  discuss child’s overweight or obesity |  |  |  |  |  |
| 20. Concern about parent’s reaction is a  barrier to discussing child’s  overweight or obesity |  |  |  |  |  |
| 21. Futility or lack of effective  intervention is a barrier to discussing overweight or obesity |  |  |  |  |  |

With children with obesity/overweight how often do you:

🞎

🞎

🞎

🞎

🞎

🞎

🞎

1. Evaluate medical conditions
2. Address nutrition
3. Address physical activity
4. Address screen time
5. Address sleep
6. Ask permission
7. Ask which lifestyle issues are most important

🞎

1. Assess readiness for change

🞎

1. Assess confidence
2. Schedule a return visit to you to address weight

🞎

🞎

1. Refer to a dietitian
2. Refer to another specialist or program for weight management

🞎

1. Give a handout with information
2. Recommend an online tool

Never Rarely

🞎

🞎

🞎

🞎

🞎

🞎

🞎

🞎

🞎

🞎

🞎

🞎

🞎

Every once in a while

🞎

Some-

times Always

🞎

🞎

🞎

🞎

🞎

🞎

🞎

🞎

🞎

🞎

🞎

🞎

🞎

🞎

🞎

🞎

🞎

🞎

🞎

🞎

🞎

🞎

🞎

🞎

🞎

🞎

🞎

🞎

🞎

🞎

🞎

🞎

🞎

🞎

🞎

🞎

1. After you refer a child to a dietitian, another specialist or program for weight management, do you usually schedule a follow-up visit to see you for overweight/obesity?

🞎

🞎

🞎

🞎

🞎

🞎

🞎

🞎

🞎

🞎

🞎

🞎

🞎

- - Follow-up visit 3 months or less after specialty visit or program
  - Follow-up visit more than 3 months after specialty visit or program
  - No planned follow-up visit

Please rate your confidence in your ability to:

1. Determine whether a child has overweight or obesity
2. Calculate body mass index

🞎

🞎

🞎

Not at all confident

🞎

Not

Confident Confident

🞎

🞎

🞎

🞎

🞎

Very Confident

children’s risk for overweight/obesity

🞎

🞎

🞎

🞎

🞎

🞎

🞎

🞎

🞎

🞎

40. Identify eating behaviors that increase

39. Interpret body mass index in children 🞎

increase children’s risk for

🞎

🞎

🞎

🞎

🞎

🞎

🞎

42. Identify physical activity barriers that

41. Counsel children and families about eating behaviors that increase children’s risk for overweight/obesity

🞎

children’s risk for overweight/obesity

🞎

🞎

🞎

🞎

overweight/obesity

1. Counsel children and families about physical activity behaviors that increase children’s risk for overweight/obesity
2. Identify sleep behaviors that increase
3. Council children and families about

🞎

🞎

🞎

🞎

sleep behaviors that increase children’s

risk for overweight/obesity

1. Identify screen time behaviors (TV, video, computer) that increase children’s risk for overweight/obesity

🞎

🞎

🞎

🞎

🞎

🞎

🞎

🞎

1. Counsel families about screen time behaviors (TV, video, computer) that increase risk for overweight/obesity
2. Identify parenting practices that

increase children’s risk for

🞎

🞎

🞎

🞎

overweight/obesity

1. Counsel families about parent practices that increase children’s risk for overweight/obesity

🞎

🞎

🞎

🞎

🞎

🞎

🞎

🞎

1. Setting behavioral goals with children and families to prevent or address overweight/ obesity
2. Using brief motivational interviewing techniques with children and families

🞎

🞎

🞎

🞎

How often is each of the following a barrier to scheduling visits specifically to address overweight or obesity?

🞎

🞎

🞎

🞎

🞎

🞎

🞎

🞎

🞎

🞎

🞎

🞎

🞎

🞎

🞎

🞎

🞎

🞎

🞎

🞎

🞎

🞎

🞎

🞎

🞎

🞎

🞎

🞎

| Never | | Rarely | Every  once in a  while | Some- times | Always |
| --- | --- | --- | --- | --- | --- |
| 52. Lack of reimbursement for a visit |  |  |  |  |  |
| 53. Lack of time during visit slot for lifestyle counseling |  |  |  |  |  |
| 54. “No show” rate is higher for visits for  overweight/obesity than for other diagnoses |  |  |  |  |  |
| 55. Families do not believe their child has overweight or obesity |  |  |  |  |  |
| 56. Families choose not to schedule visits for overweight/obesity even when they  recognize child’s high weight |  |  |  |  |  |
| 57. Your belief that visits for  overweight/obesity are not worthwhile |  |  |  |  |  |
