# Supplementary material for: Implementation science evaluation of an eHealth pediatric primary-care overweight and obesity intervention using the RE-AIM evaluation framework
Source: PLoS One. 2026 Feb 9;21(2):e0341635. doi: 10.1371/journal.pone.0341635 (PMC12885277; doi:10.1371/journal.pone.0341635)
Supplement: S4 Appendix — Dynamo Kids! Patient Discussion Summary. (DOCX) [file pone.0341635.s003.docx]

**Dynamo Kids! Patient Discussion Summary**

| Patient Initials | COPC: *(circle)*  DeHaro Hatcher Southeast |
| --- | --- |
| Date of Visit |  |

| **OTHER/COMMENTS** | | |
| --- | --- | --- |
| **Topic(s) discussed:** | *Check if you asked/discussed*  **Use of Dynamo Kids**  **Whether changes in health behavior(s) were made**  **Child’s health risks**  **Growth tracking** |  |
| **Parent goal(s) for after the visit**  DK site suggests parents set goals in 3 topics   - days w/ no sugar-sweetened beverage - daily minutes of physical activity - evening meals each week based on MyPlate | **Yes discussion of goals after visit**  **No discussion of goals after the visit** | **If yes,** *check parent goals*  Continue current goal(s) *(circle all that apply)*  Sugar-sweetened beverages Physical activity MyPlate    Set 1 or more new goal(s) *(circle all that apply)*  Sugar-sweetened beverages Physical activity MyPlate    Other__________________    None (parent decided no goals) |
| **Praise/motivational interviewing used**  E.g.: you acknowledged parent challenges, praised priorities and efforts, expressed confidence) | **Yes**  **No** |  |
| **Any next steps** | **Yes**  **No (except routine well child visit)** | **If yes** *(check one or more)*  Follow-up visit with this provider for weight health  Resources (handouts) provided  Dietitian referral  Specialist referral  Other ____________ |

***Please give to nurse manager to return to Dynamo Kids! team.***
