# Supplementary material for: Implementation science evaluation of an eHealth pediatric primary-care overweight and obesity intervention using the RE-AIM evaluation framework
Source: PLoS One. 2026 Feb 9;21(2):e0341635. doi: 10.1371/journal.pone.0341635 (PMC12885277; doi:10.1371/journal.pone.0341635)
Supplement: S5 Appendix — Website. (DOCX) [file pone.0341635.s004.docx]

Appendix 5. Posttest Only: End of Intervention Parent-Reported Behavioral Changes

| **Post-Intervention Sugar-Sweetened Beverages Behaviors** | n (%) |
| --- | --- |
| *How much juice is consumed?* |  |
| Less than 1 cup a day | 20 (51.28) |
| More than 1 cup a day | 19 (48.72) |
| *How much soda is consumed?* |  |
| Less than 1 cup a day | 31 (77.50) |
| More than 1 cup a day | 9 (22.50) |
| *How much sweet tea is consumed?* |  |
| Less than 1 cup a day | 34 (85) |
| More than 1 cup a day | 5 (15) |
| *How much water is consumed?* |  |
| Less than 2 cups a day | 2 (5.71) |
| More than 2 cups a day | 33 (94.29) |
| **Post-Intervention *Physical Activity (PA)*** **Behaviors** |  |
| *Number of Days Child Does PA During the Week* |  |
| More than 2 days a week | 24 (68.57) |
| Less than 2 days a week | 11 (31.43) |
| *Amount of Daily PA During Weekdays* |  |
| Does not Engage | 0 (0) |
| Engages less than an hour | 28 (80) |
| Engages more than an hour | 7 (20) |
| *Number of Days Child Does PA During Weekend* |  |
| Does not engage | 4 (11.43) |
| 1 day | 24 (68.57) |
| 2 days | 7 (20) |
| *Amount of Daily PA During Weekend* |  |
| Does not engage | 1 (2.86) |
| Engages less than an hour | 20 (57.14) |
| Engages more than an hour | 14 (40) |
| **Post-Intervention MyPlate Behaviors** |  |
| How often during the week are MyPlate meals prepared |  |
| None | 3 (9.68) |
| 1-2 times | 9 (29.03) |
| 3-4 times | 13 (41.94) |
| 5 or more times | 6 (19.35) |
